# Dielectrophoresis of gold nanoparticles conjugated to DNA origami structures

**DOI:** 10.3762/bjnano.7.87

**Published:** 2016-07-01

**Authors:** Anja Henning-Knechtel, Matthew Wiens, Mathias Lakatos, Andreas Heerwig, Frieder Ostermaier, Nora Haufe, Michael Mertig

**Affiliations:** 1 Physikalische Chemie, Mess- und Sensortechnik, Technische Universität Dresden, 01062 Dresden, Germany; 2 Department of Biology, New York University Abu Dhabi, Abu Dhabi, UAE; 3 Department of Chemistry, University of Alberta, Edmonton, T6G2G2, Canada; 4 Kurt-Schwabe-Institut für Mess- und Sensortechnik Meinsberg e.V., 04736 Waldheim, Germany

**Keywords:** gold nanoparticles, dielectrophoresis, DNA nanotechnology, DNA origami, self-assembly

## Abstract

DNA nanostructures are promising construction materials to bridge the gap between self-assembly of functional molecules and conventional top-down fabrication methods in nanotechnology. Their positioning onto specific locations of a microstructured substrate is an important task towards this aim. Here we study manipulation and positioning of pristine and of gold nanoparticle-conjugated tubular DNA origami structures using ac dielectrophoresis. The dielectrophoretic behavior was investigated employing fluorescence microscopy. For the pristine origami, a significant dielectrophoretic response was found to take place in the megahertz range, whereas, due to the higher polarizability of the metallic nanoparticles, the nanoparticle/DNA hybrid structures required a lower electrical field strength and frequency for a comparable trapping at the edges of the electrode structure. The nanoparticle conjugation additionally resulted in a remarkable alteration of the DNA structure arrangement. The growth of linear, chain-like structures in between electrodes at applied frequencies in the megahertz range was observed. The long-range chain formation is caused by a local, gold nanoparticle-induced field concentration along the DNA nanostructures, which in turn, creates dielectrophoretic forces that enable the observed self-alignment of the hybrid structures.

## Introduction

The DNA origami method facilitates high throughput synthesis of identical and fully addressable two- (2D) or three-dimensional (3D) nanoscaled structures [1–3]. Such DNA constructs constitute promising template structures to combine different organic and inorganic nanomaterials into tailored hybrid nanodevices, and hence, hold the potential to combine self-assembly of functional molecules with conventional fabrication methods in nanotechnology [4–11]. To this aim, one important task is the precise positioning and alignment of DNA structures on technically patterned surfaces, e.g., their controlled deposition into microstructured electrode arrays. Recent studies address the alignment of DNA nanostructures to pre-structured surfaces that include (i) notches with the shape and dimension corresponding to the DNA origami structure [12–13], or (ii) gold islands to align DNA nanostructures between two conducting pads [14]. An alternative route is the alignment of the DNA origami structures within a microelectrode contact array through hydrodynamic flow [15] or – with higher selectivity – by using dielectrophoresis (DEP) [16–19].

DEP is an electrokinetic phenomenon and results in a force that moves polarizable objects either towards regions with the highest (positive DEP (pDEP)) or lowest electrical field gradient (negative DEP). The direction of the dielectrophoretic force depends on the properties of the applied ac electrical field, e.g., frequency and amplitude, and physical parameters of the object and its surrounding media, e.g., conductivity and polarizability. DEP has been investigated for the spatial manipulation of various nanomaterials such as carbon nanotubes, proteins and nucleic acids [17–18,20–27]. Recently, it has been shown that DNA origami structures can be dielectrophoretically trapped along an electrode structure using frequencies starting from 1 MHz at an electrical field strength of about 1·10^7^ V/m [28–30]. A more precise deposition of 2D single layer and 3D multilayer DNA constructs required a higher frequency of approximately 12.5 MHz. This frequency range can be lowered when insulator-based dielectrophoresis is used [19]. In detail, an array of polydimethylsiloxane (PDMS) pillars influences the field gradients between two electrodes. This results in an inhomogeneous field around each post that enables multiple trapping of 2D as well as 3D hollow DNA origami structures using frequencies around 1.5 kHz and an electrical field strength between 5·10^4^ V/m and 2.1·10^5^ V/m.

Herein we show that DNA origami trapping can be considerably influenced by the attachment of nanometer-sized, polarizable particles that act as supported floating nanoelectrodes, and thus, as field concentrators.

## Results and Discussion

**Figure 1:**
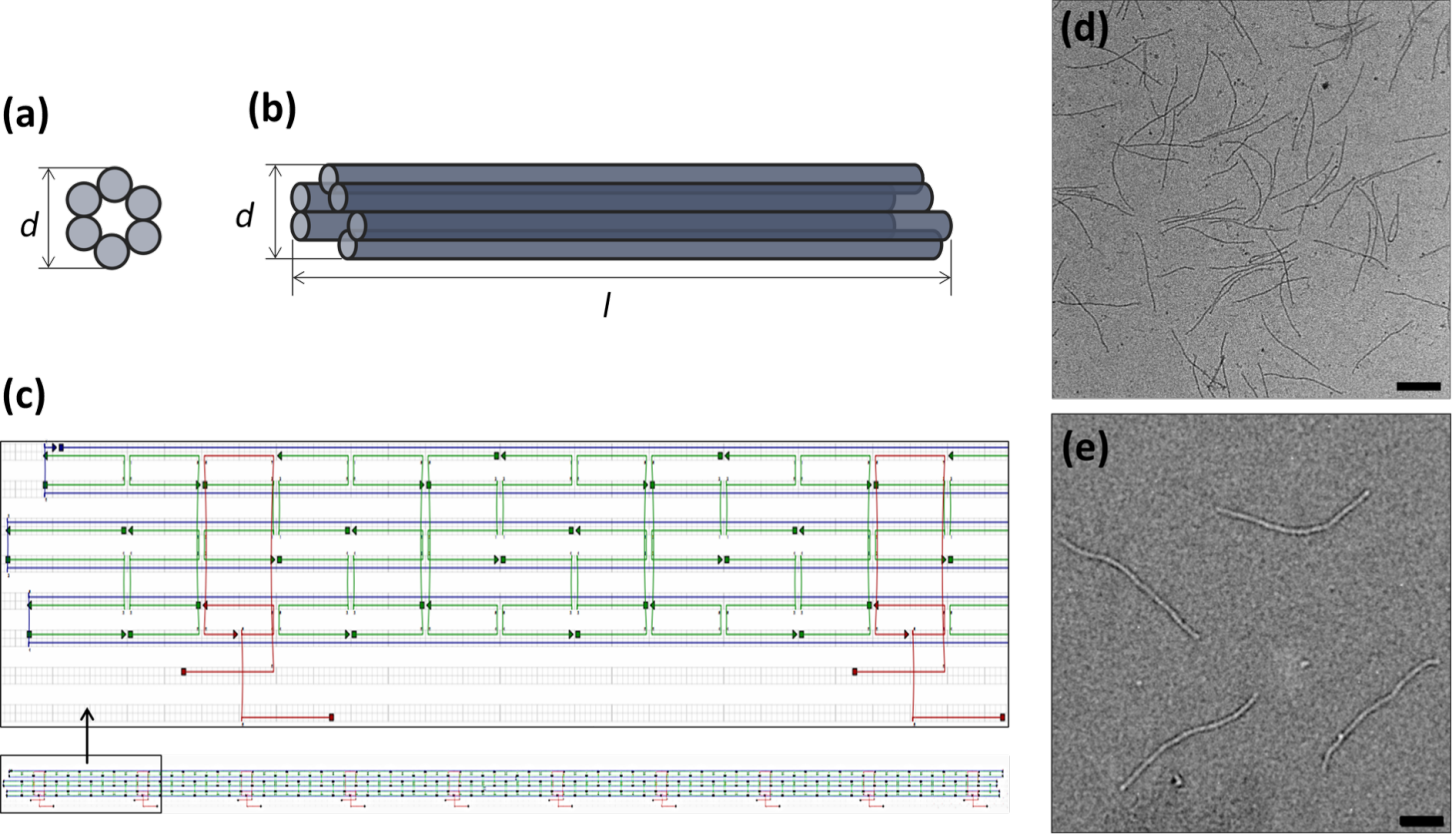
Six-helix bundle. The (a) front and (b) side view of the six-helix bundle are schematically depicted (diameter: *d* = 6 nm, length: *l* = 414 nm). (c) caDNAno design representation with the template strand in blue, and main and capture staples in green and red, respectively. The capturing strands are 126 bases apart from each other on both helices. (d,e) TEM images of the resulting tubular DNA structures. The scale bar is (d) 200 nm and (e) 100 nm.

For our studies, we focused on a 414 nm long, tubular DNA-origami structure, a so-called six-helix bundle (6HB, Figure 1). The diameter is approximately 6 nm. For the assembly, the single-stranded circular *M13mp18* DNA scaffold strand is folded with staples in a z-shaped pattern into six, parallel arranged double helices. Our design includes ten particle-binding sites, each consisting of two single-stranded nucleic acid overhangs with a poly(A) sequence that hang out from the 6HB along two adjacent double helices of the origami. The distance between neighboring particle binding sites is 42 nm.

**Figure 2:**
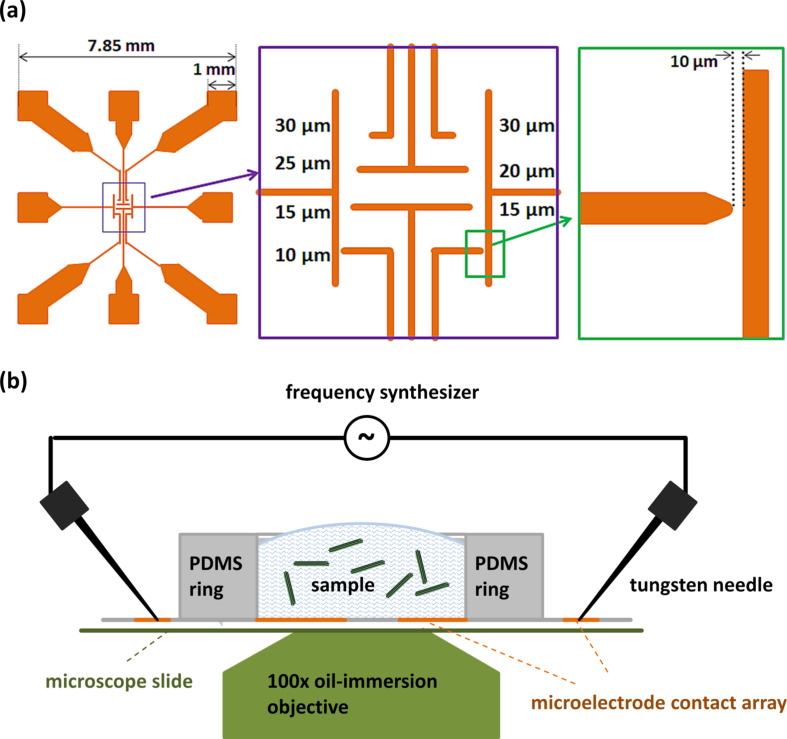
Experimental set-up for the investigation of the dielectrophoretic trapping. Schematic image of the (a) microelectrode contact array and (b) experimental set-up.

**Figure 3:**
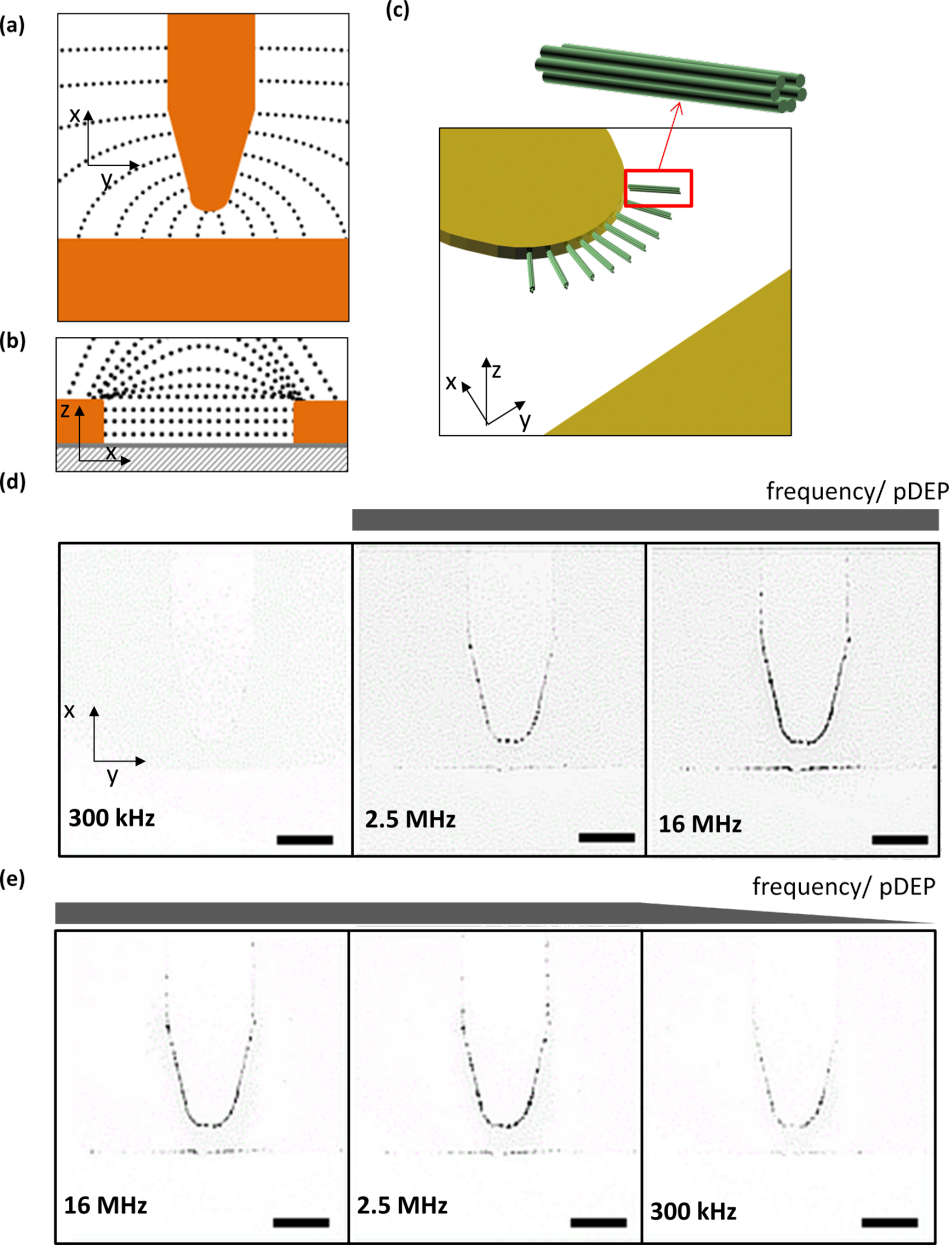
Dielectrophoretic manipulation of six-helix bundles. Schematic representation of the (a) top and (b) side view of the field line gradient, and (c) the six-helix bundle trapping by positive DEP. Potential is applied along the x-axis. Inverted fluorescence microscopy images of the measurement series taken at 1·10^6^ V/m with a stepwise (d) increasing or (e) decreasing frequency. The scale bar is 25 μm.

The dielectrophoretic behavior of the 6HBs was studied using a microelectrode contact array with eight electrode pairs. As shown in Figure 2a, one electrode is in the form of a rounded microtip that points towards a rectangular electrode. This results in an asymmetric electrical field along the x- and y-directions with a higher field gradient around the tip electrode (Figure 3a). Thus, the motion of the DNA origami structures, driven by pDEP, is well defined. The gold electrode array was fabricated by photolithography on a glass cover to monitor the motion and positioning of the YOYO^®^-1 stained 6HB by using fluorescence microscopy. The electrode pairs with the smallest distance of 10 μm were chosen for all performed experiments in order to obtain a high electrical field. A PDMS ring (height = 1 mm, *d*_i_ = 2 mm, sealed with silicon oil) was mounted around the electrode array, representing the electrode chamber. As depicted in Figure 2b, the electrode pair of interest was electrically contacted outside the liquid volume with tungsten needles that are connected to a frequency and voltage synthesizer.

DEP is influenced by the complex permittivity of the manipulating object (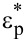) and its surrounding medium (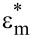). This parameter is at low and high frequencies a function of the electrical conductivity, σ, and the dielectric constant, ε, respectively [31]. It applies that for pDEP 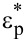 has to be larger than 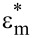. Since the ε of water is approximately 8-fold larger than that of DNA, we expect pDEP only to occur in the σ-dominated range at σ_p_ >> σ_m_. In order to ensure this condition, the as-prepared 6HB solution was diluted with distilled water to a final concentration of approximately 45 pM. The final concentration of Mg^2+^ ions was approximately 1.5 mM, which we found was sufficient to prevent the disassembly of the DNA origami structure.

We conducted measurement series with frequency and potential being swept. Details are explained next. In the first series, the potential was set to 0.1 V and the frequency was increased starting from 100 kHz to 16 MHz in steps of 100 kHz. In the second series, the potential was increased to 0.2 V, and the same frequency sweep was applied; these series configurations were perused until 10 V.

Regarding the change in potential, we found that the 6HB started to move into the increasing field gradient, and thus, underlying pDEP, at a rather high threshold electrical field strength of 5·10^5^ V/m. A further increment of the voltage resulted in a higher density of trapped DNA origami structures around the rim of the tip electrode. Figure 3d and Figure 3e show a selection of the microscopy images of the measurement series taken at 1·10^6^ V/m. We did not observe any pDEP for frequencies in the kilohertz range. A frequency of at least 2.5 MHz was required to arrange the DNA nanotubes at the electrode edges, where the number of trapped 6HBs increased with longer duration of the applied field. A frequency dependence in the σ-dominated range with a trapping minimum in the upper kilohertz range was also observed before for DNA molecules and attributed to an ac electro-osmotic flow that moves the DNA molecules above the electrode surface, outside of the microscopic field of view [27].

The positioning of the tubular DNA nanostructures was observed to primarily take place at the tip electrode due to the highest field gradient there. However, a small proportion of the 6HBs was also observed to be deposited at the oppositely located, rectangular electrode, which is due to an additional field gradient along the edge of the electrode tip in the z-axis direction, as depicted in Figure 3b. Turning the field off resulted in immediate diffusion of the 6HBs away from the electrodes confirming that the trapping only occurs in the presence of an electrical field.

**Figure 4:**
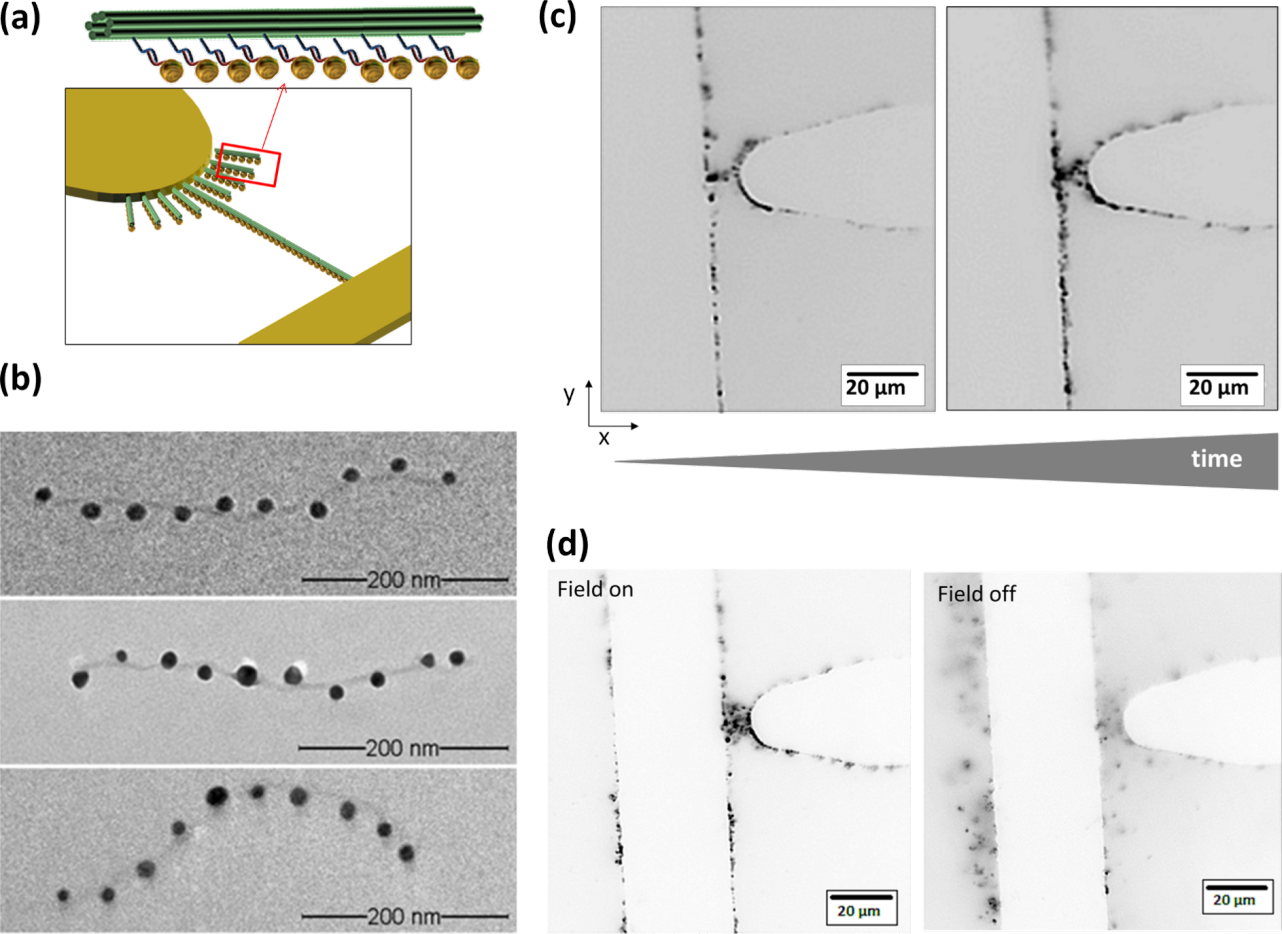
Dielectrophoretic manipulation of gold nanoparticle-conjugated six-helix bundles. (a) Schematic representation of the positive DEP manipulation. (b) TEM images of gold nanoparticle-modified six-helix bundles. (c) Resulting DNA origami structure arrangement as a function of time at 1·10^6^ V/m and 16 MHz. Inverted fluorescence microscopy image after 16 min (left) and 26 min (right) ac field application. (d) Inverted fluorescence microscopy images of the trapping behavior during on/off-switching of the ac field.

We then conjugated 15 nm gold nanoparticles to oligonucleotides with a poly(T) sequence, and further attached them to the ten double-sticky-end locations along the DNA nanostructure through hybridization. Figure 4b shows transmission electron microscopy (TEM) images of representative functionalized DNA origami structures. The resulting structures contained eight to ten gold nanoparticles at the sticky-end locations along the 6HBs with a yield of 89%.

The experiments with voltage and frequency sweeps demonstrate that the tubular DNA/gold-nanoparticle hybrid structure possesses a different dielectrophoretic response than its unmodified counterpart. Trapping at the electrodes edges was observed at a lower electrical field strength (1·10^5^ V/m) and lower frequencies in the kilohertz range (e.g., 500 kHz with 1·10^6^ V/m). As for the pristine 6HBs, the number of observed DNA origami structures at the rims of the electrodes increases with longer duration of the field application (data not shown).

The change in the frequency dependency of the dielectrophoretic trapping of gold nanoparticle-conjugated and of pristine DNA origami can be explained by the difference in polarizability of the metallic nanoparticles and the DNA nanostructure, i.e., the dipole relaxation time and the nature of the dipole. A dipole in gold nanoparticles is induced due to direct polarization of the electron cloud, whereas polarization of a negatively charged DNA molecule is proposed to be due to a combination of its surrounding and mobile counter ions [32]. A physical measure of the polarizability is the dielectric constant, ε. Experimental data show that DNA molecules with ε = 8.5 are a less polarizable material than gold nanoparticles (ε → ∞) [33–34]. Hence, the higher polarizability of gold nanoparticles extends the electrical field strength and frequency range of DNA/gold-nanoparticle hybrid structures for pDEP [35–36].

Furthermore, the gold-nanoparticle modification resulted in another interesting observation: At an electrical field strength of 1·10^6^ V/m and a frequency of 16 MHz, the 6HBs started to linearly arrange into chain-like structures beginning from the tip electrode towards the plate electrode (Figure 4c). With increasing duration of the field application, the chain formation intensified while further DNA origami chains may occur around the field intensity maxima. Switching the field off resulted in diffusing of the structures away from the electrodes, and thus, disassembling of the chains (Figure 4d). On the one hand, this is a clear sign that the gold nanoparticle-conjugated DNA origami do not irreversibly aggregate during DEP deposition, as it was observed for the formation of conducting wires by DEP deposition of plain, unsupported gold nanoparticles [35–39]. On the other hand, the disassembly of our chain structures upon switching off the electrical field prohibits any further high-resolution investigation of the arrangement, i.e., by TEM.

It has been shown that the interaction between ac field-induced dipoles in plain, unsupported gold nanoparticles results in the formation of stable gold nanoparticle assemblies [35–39]. The growth of bifurcated gold nanoparticle chains from nanoparticle suspensions by DEP has been reported between 10 to 200 Hz for up to 4·10^4^ V/m [35–36], between 10 kHz and 1 MHz for about 2.5·10^6^ V/m [37] or above 1.3·10^7^ V/m [38], and between 1 MHz and 10 MHz for 1·10^7^ to 5·10^8^ V/m [39]. In our experiments, we never observed an irreversible formation of stable nanoparticle wires. We suppose that this is due to two reasons. First, the DNA origami, to which the nanoparticles are attached, keeps them at a distance that does not facilitate direct contact between them to aggregate. TEM imaging revealed a gap size between the gold nanoparticles of 26 ± 5 nm (mean ± s.e.m., *n* = 36). Second, after the incubation of the 6HB with gold nanoparticles, the excess of unbound gold nanoparticles was efficiently removed by gel electrophoresis. Instead, in our experiments the observed structures were, compared to the reported gold-nanoparticle chains, subject to disassembly in the absence of the electrical field [36]. In addition, we also did not observe branching of the assembled chains, which is typical for plain gold-nanoparticle chains, which is due to the simultaneous trapping of two gold nanoparticles at the tip of the growing chain. We found a self-aligned, linear growth of the structure from the tip towards the plate electrode for the DNA origami when conjugated with gold nanoparticles, indicating that the gold nanoparticles are indeed well separated due to their attachment along the supporting DNA origami.

We hypothesize that the linear formation of the gold nanoparticle-conjugated structures is facilitated by (i) the collectively higher dielectrophoretic response of the origami-supported gold nanoparticles, and (ii) the generation of local fields of higher intensity and steeper gradient around them.

The first effect is related to the phenomenon that the induced dipole moments tend to align parallel to the electrical field lines, which –for the complete hybrid structure– is achieved, when the longer origami is oriented in parallel to the field lines. This will lead to a determining deposition directionality perpendicular to the electrode edges.

**Figure 5:**
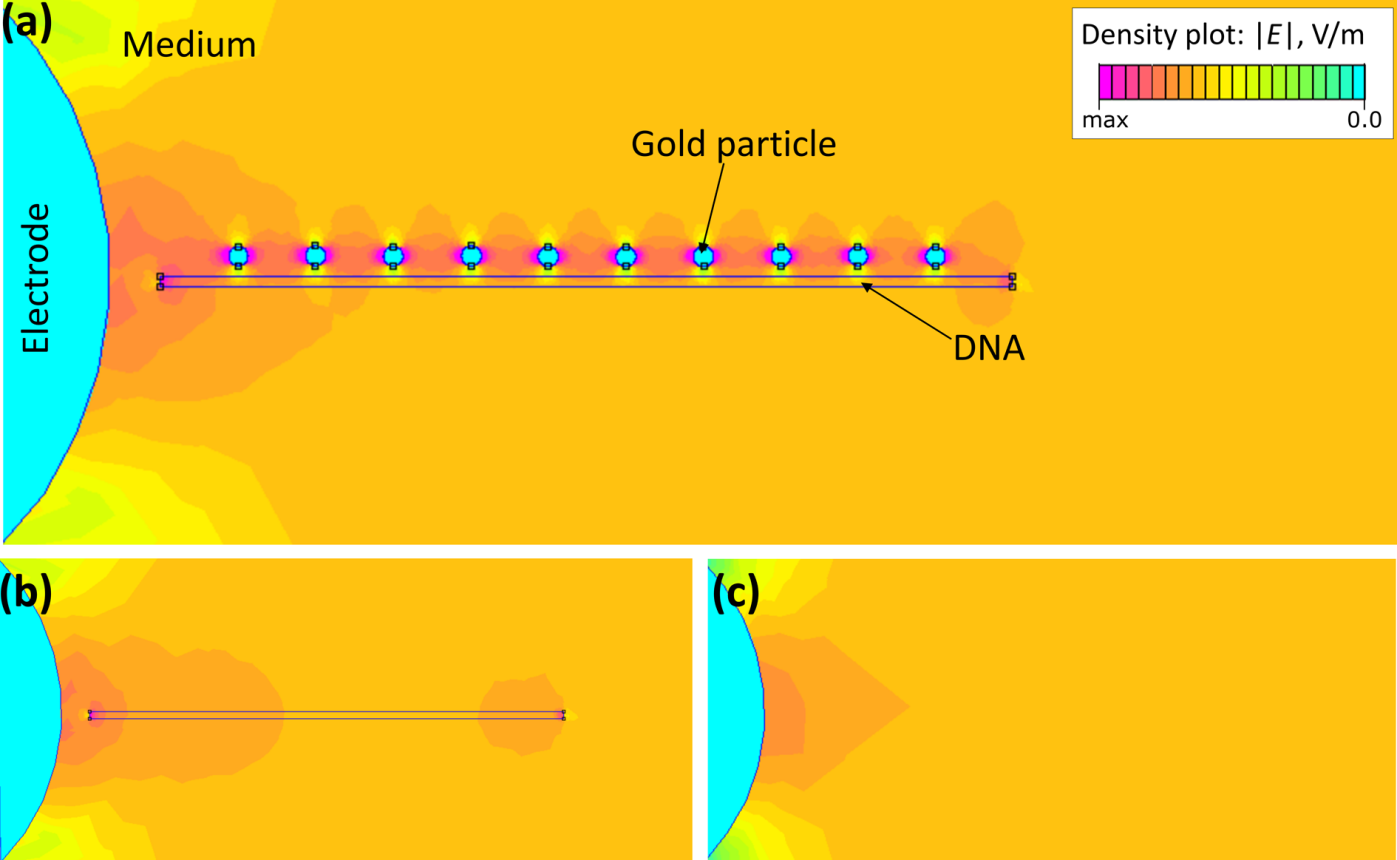
Electrical field intensity (a) in the presence of a gold-nanoparticle modified DNA structure, (b) plain DNA structure at the left electrode, and (c) absence of a DNA nanostructure as obtained from finite element method simulations.

The second reason for the observed self-alignment is expected to be related to the fact that an origami-supported gold nanoparticle acts as floating electrode. The use of floating electrodes for the local enhancement and manipulation of DEP has been intensively studied [40–43]. Without the need for increasing the applied voltages, these studies have shown that floating nanoelectrodes increase local DEP forces by orders of magnitudes. In the floating-electrode DEP (feDEP) approach, a local non-uniform field is obtained by introducing a passive, metallic element into an imposed field. The passive element responds to the imposed field by capacitive coupling, which in turn, causes a local alteration of the electrical field, and thus, an enhancement of the field gradient. The main difference between studies described in [40–43] and our investigations is that in the literature the passive elements are written lithographically from the beginning, whereas in our case the floating elements are supported by the origami structure and are deposited in between the actively driven electrodes only within the DEP deposition process itself. Figure 5a shows a model calculation of the field distribution for the case that one gold-nanoparticle/DNA-origami hybrid structure is deposited to the edge of an electrode. It is clearly seen that the supported nanoparticles act as a linearly arrayed ensemble of floating electrodes that will focus the field lines along the supported nanoparticles. Here, the gold nanoparticle ensemble enables an extension of the dense field lines towards the furthermost gold nanoparticle, and thus, towards the electrode gap, facilitating a preferred deposition of the next hybrid structure at this specific site, and thus, chain growth. Figure 5b shows that self-alignment will not happen, when pristine origami structures are used.

## Conclusion

We have shown that the dielectrophoretic behavior of DNA origami structures can be influenced with the attachment of polarizable nanoelements. In detail, the tubular gold nanoparticle-conjugated DNA origami exhibits pDEP over a larger frequency range than its plain counterpart, and forms a linear arrangement at megahertz frequencies. The observed long-range self-alignment effect is presumably caused by the action of the DNA-origami-supported nanoparticles as floating electrodes, enhancing the local DEP forces. We believe that this finding gives an alternative route for the construction of higher-order arrangements of DNA nanostructures. The DEP-based deposition of gold nanoparticle-functionalized DNA origami structures might be particularly suitable for the fabrication of long-distance plasmonic waveguides that are difficult to realize with lithographic methods such as e-beam lithography or AFM manipulation [44]. The different behavior of modified and unmodified origami structures can be also useful for the fast verification of DNA origami modifications as well as separation of different species of DNA nanostructures within a microfluidic device.

## Experimental

### Preparation of the DNA origami structures

The 6HB was designed using the software caDNAno [45]. Staple strands and the single stranded scaffold *M13mp18* were purchased from Life Technologies and Bayou Biolabs, respectively. For the assembly, the 50 nM staple strands were combined with the 10 nM template in a Mg^2+^ containing 1×TE buffer solution (12.5 mM MgCl_2_, 40 mM Tris, 2 mM EDTA, pH 8.1). Then, the mixture was heated to 85 °C for 15 min, cooled to 56 °C and further cooled to 4 °C after 1 h. In order to purify the resulting structure from excess staple strands, the sample was spin filtered at 2300*g* with an Amicon 100 kDa MWCO filter device (Millipore) according to the manufacturer's instructions and washed thrice with 1×TE buffer solution.

### Preparation of gold nanoparticles

Spherical gold nanoparticles with 15 nm in diameter were prepared following a procedure of De Mey [46]. This method is based on an inverse order of reactant addition compared to the classical citrate method. The initial solution of 106 mL of 2.2 mM sodium citrate was brought to boil under stirring before 1 mL of 24.3 mM gold chloric acid (HAuCl_4_) was rapidly added. Stirring and heating was continued for 30 min until the solution color did not change anymore.

### Preparation of gold nanoparticle-conjugated DNA origami structures

For the preparation of DNA-coated gold nanoparticles [47–52] we first phosphinated the surface of the gold nanoparticles as follows: 0.4 mg of bis(*p*-sulfonatophenyl)phenylphosphine dihydrate dipotassium salt (BSPP) were added to 1 mL gold nanoparticle solution and mixed for 48 h. Solid NaCl was dissolved in this solution until a color change from pinkish to light purple occurred. Then, the solution was spun down at 7500*g* for 30 min and the supernatant carefully removed. The remaining pellet was resuspended in a mixture of 100 µL of 2.5 mM BSPP and 100 µL of methanol. The solution was once more spun down to dissolve the pellet in 2.5 mM BSPP. The gold nanoparticles were further modified with oligonucleotides that included a thiol functionality at the 3′ end (sequence: ^5′^TTTTT TTTTT TTTTT TTT–C_3_H_5_SH^3′^, HPLC purified, Biomers.net). For this, 3.2 pmol phosphinated gold nanoparticles were mixed in equivalent ratio with the oligonucleotides in Na^+^-TBE buffer solution (50 mM NaCl, 89 mM Tris, 89 mM boric acid, 1 mM EDTA pH 8.0) and shaken for 12 h at room temperature.

After this incubation the gold nanoparticle solution was centrifuged 3 times at 5000*g* for 15 min, to get rid out the excess of unbound staple strands. The particle attachment to the 6HBs was done at a ratio of 100:1 (nanoparticle/six-helix bundle) at room temperature for at least 12 h in 1×TE buffer with 12.5 mM MgCl_2_ and 300 mM NaCl.

Finally, excess of gold nanoparticles was removed by gel electrophoresis (0.6% agarose gel in 0.5×TAE buffer with 5 mM MgCl_2_, 2 h, 80 V) and Freeze `N` Squeeze purification of the separated band for the gold-nanoparticle modified DNA origami.

### Transmission electron microscopy

TEM images of the DNA structures were taken with a Zeiss Libra 200MC operated at an accelerating voltage of 200 kV and performed on copper/formvar/carbon grids (400 mesh, 3.05 mm diameter, Plano GmbH). Before deposition, the grids were glow discharged for 30 s using a SPI supplies Plasma prep II machine. A volume of 10 µL of the DNA origami structures was adsorbed on the grid for 5 min and wicked away. Then, a 0.1% uranyl formate solution (5 µL) was added for 5 min and again wicked away. Finally, the grid was gently washed with double-distilled water and left to dry for 10–15 min before imaging.

### Preparation of the microelectrode contact array

Micro-patterned gold electrodes were prepared on glass slides by optical lithography. For this, the glass slides (16 × 16 mm, 0.13–0.16 mm thick) were first cleaned in a piranha solution [30 mL H_2_SO_4_ (95–97%), 10 mL H_2_O_2_ (30%)] for 10 min, then rinsed with double-distilled H_2_O (ddH_2_O) and blow dried under a nitrogen stream. 80 µL of the positive resist AR-P 5350 (Allresist) was spin-coated (4500 rpm/s, 4500 rpm, 30 s) on the glass slides and heated at 105 °C on a hotplate for 5 min. A lithographic photomask was placed and the construct exposed for 2.5 min to UV light (365 nm). For the development of the photoresist, the glass slides were incubated for 30–60 s into a developer solution (AR 500-47, Allresist; 1:2 dilution in ddH_2_O). An adhesion layer of 3 nm chrome and 30 nm gold was thermally evaporated and finally the photoresist was removed with the remover solution AR 300-72 (Allresist) by sonication.

### Dielectrophoretic manipulation of the 6HBs

The gold pads were cleaned by immersing them stepwise for 20 s into 100% fuming nitric acid (Merck) and 1 min into a neutralization solution [hydrogen peroxide (30 wt % in water; Merck), ammonia solution (25 wt % in water; Merck) and ddH_2_O in the ratio 1:1:5] and rinsed with ddH_2_O. Then, such a glass slide was placed in an inverted optical microscope (Axiovert 200M, Carl Zeiss MicroImaging) equipped with a 100×/1.45 numerical aperture oil immersion objective and appropriate fluorescence filter sets. A PDMS ring was sealed with silicon oil on the glass substrate in between the electrodes and contact pads. In addition, the microscope was equipped with micromanipulators (Suess MICROTec PH100) that were used to place tungsten needles (SIGNATONE SE-T) on the contact pads, and thus, connect the electrodes with the function generator (Textonix AFG 320; Sony). The DNA nanostructures were stained with YOYO^®^-1 (Life Technologies) in a ratio of 1:10 and diluted with ddH_2_O to a final concentration of 45 pM 6HBs and 1.5 mM Mg^2+^. 15 µL of this solution was pipetted into the PDMS ring and an (ac) field applied. Images were taken in the green channel with a frame-transfer intensified CCD camera (Cascade 512:B, Roper Scientific) using the MetaMorph software (Molecular Devices).

### Finite element method simulations

For the two dimensional visualization of the gold nanoparticles influence on the electrical field the software package FEMM4.2 (http://www.femm.info/wiki/HomePage) was used. For the simulation, a dielectric constant of 8.5 and 78 was used for DNA and the surrounding medium, respectively.

## Supporting Information

File 1 Sequences of staple strands.
