# Supplementary material for: Dielectrophoresis of gold nanoparticles conjugated to DNA origami structures
Source: Beilstein J Nanotechnol. 2016 Jul 1;7:948–56. doi: 10.3762/bjnano.7.87 (PMC4979641; doi:10.3762/bjnano.7.87)
Supplement: File 1 — Sequences of staple strands. [file Beilstein_J_Nanotechnol-07-948-s001.pdf]

Supporting Information

for

Dielectrophoresis of gold nanoparticles conjugated to DNA origami structures

Anja Henning-Knechtel*[1,2], Matthew Wiens[1,3], Mathias Lakatos[1], Andreas Heerwig[1,4], Frieder Ostermaier[1], Nora Haufe[1,4] and Michael Mertig*[1,4]

Address: [1]Physikalische Chemie, Mess- und Sensortechnik, Technische Universität Dresden, 01062 Dresden, Germany; [2]present address: Department of Biology, New York University Abu Dhabi, Abu Dhabi, UAE; [3]Present address: Department of Chemistry, University of Alberta, Edmonton, T6G2G2, Canada and [4]Kurt-Schwabe-Institut für Mess- und Sensortechnik Meinsberg e.V., 04736 Waldheim, Germany

Email: Anja Henning-Knechtel* - anja.henning@nyu.edu;

Michael Mertig* - michael.mertig@tu-dresden.de

* Corresponding author

Sequences of staple strands

Number	Sequence of staples (5 prime to 3 prime)
1	TGGTCAATAACCTGTCTAAAATATCTTTAGGTGAGGCGGTCA
2	GAACGAGTAGATTTCCGTCAATAGATAACATTAAAAATACCG
3	TGTAGCTCAACATGACGTTATTAATTTTAAAGCGTAAGAATA
4	ATTTTTGCGGATGGGGAACAAAGAAACCAAGGGACATTCTGG
5	GCTTCAAAGCGAACTTGTTTGGATTATATGGAAATACCTACA
6	AGAGGAAGCCCGAAACCATATCAAAATTGAACAATATTACCG
7	AACGAGAATGACCAACAGTACCTTTTACTTGTAGCAATACTT
8	ATATTCATTGAATCGATTGCTTTGAATAGGCCACCGAGTAAA
9	GCTTTTGCAAAAGAAACATCAAGAAAACCAAGAATTGAGTGG
10	CCCTCGTTTACCAGTGAATTACCTTTTTAGTCAGAGGGTAAT
11	GATTTAGGAATACCTAATTTTCCCTTAGTGAAAATAGCAGCC
12	AACGGAACAACATTATTAAGACGCTGAGCCCAATCCAAATAA
13	GAATTACCTTATGCGGTTATATAACTATTTTATCCTGAATCT
14	AAATTGGGCTTGAGAAGACAAAGAACGCCCTTAAATCAAGAT
15	ATATTCATTACCCAACCGTGTGATAAATCAAATCAGATATAG
16	GGCTGGCTGACCTTATCATAATTACTAGTTTATTTTCATCGT
17	GAGGCGCAGACGGTGCTTAATTGAGAATATCAATAATCGGCT
18	GTGTCGAAATCCGCTTTAGGCAGAGGCAAAAAATAATATCCC
19	AGCAAAATTAAGCAGCTGCATTAATGAACAACAGCTGATTGC
20	GGTTGTACCAAAAAGCGCTCACTGCCCGCAGCAAGCGGTCCA
21	AGAAGCCTTTATTTCTGGGGTGCCTAATCTGTTTGATGGTGG
22	CTCATATATTTTAAAATTCCACACAACATCAAAAGAATAGCC
23	AGATTCAAAAGGGTATGGTCATAGCTGTTGGAACAAGAGTCC
24	ATCAATATGATATTGACTCTAGAGGATCAAAGGGCGAAAAAC
25	GGAGAGGGTAGCTATGTAAAACGACGGCGAACCATCACCCAA
26	GTCATTGCCTGAGACGATTAAGTTGGGTAAAGCACTAAATCG
27	CGGTAATCGTAAAATCGCTATTACGCCAGCTTGACGGGGAAA
28	TGATAATCAGAAAAAGGCTGCGCAACTGAAGAAAGCGAAAGG
29	AAATATTTAAATTGGCTTCTGGTGCCGGGCGGTCACGCTGCG
30	ATTAAATTTTTGTTTCGGCCTCAGGAAGGCGCCGCTACAGGG
31	ACGCCATCAAAAATTCGTAACCGTGCATGTATAACGTGCTTT
32	CTTTCATCAACATTTTGACCGTAATGGGCATAAGCCCAATAA
33	CCGTAACACTGAGTCCAATAGGAACCCAAGCTATCTTACCGA
34	GTAGCATTCCACAGCCACCCTCAGAGCCGCCGAACAAAGTTA
35	AAAGTTTTGTCGTCGGTTTAGTACCGCCTAACGGAATACCCA
36	GTATGGGATTTTGCATATAAGTATAGCCTTACGCAGTATGTT
37	TGAGAATAGAAAGGTTTTGCTCAGTACCGTGGCAACATATAA
38	TTTTTCACGTTGAAAGAGGCTGAGACTCTTTATTTTGTCACA
39	GCCTTTAATTGTATTGCCTATTTCGGAAGCGCCAAAGACAAA
40	TTTCTTAAACAGCTGTGCCTTGAGTAACGAAGGTAAATATTG
41	AACAACCATCGCCCGATGATACAGGAGTTCACCGTCACCGAC
42	GGCTTGCAGGGAGTTCTCTGAATTTACCAAATCACCAGTAGC
43	CTCAGCAGCGAAAGCAAACAAATAAATCTCACCAATGAAACC
44	TACAGAGGCTTTGAACAGGAGGTTGAGGACAGAATCAAGTTT

45	TCCATTAAACGGGTGAGCCGCCACCAGATCATCGGCATTTTC
46	CAACCTAAAACGAACCGCCACCCTCAGAATCTTTTCATAATC
47	AACCTGTCGTGCCAATAAAGCCTCAGAGCATTTGGGGCGCGA
48	ACATTAATTGCGTTCATTATGACCCTGTATACATTTCGCAAA
49	ATAAAGTGTAAAGCCAACGCAAGGATAAATTCCCAATTCTGC
50	TGTTATCCGCTCACATGCAATGCCTGAGCTAAAGTACGGTGT
51	TCGAATTCGTAATCGAGAAAGGCCGGAGGCTGAATATAATGC
52	CATGCCTGCAGGTCCAACCGTTCTAGCTTTTGATAAGAGGTC
53	CCCAGTCACGACGTTTTTTGAGAGATCTACTCCAACAGGTCA
54	GATGTGCTGCAAGGGTCTGGAGCAAACAGCGTTTTAATTCGA
55	CGGTGCGGGCCTCTCTAGCATGTCAATCCATCAAAAAGATTA
56	GCCATTCGCCATTCGCCCCAAAAACAGGGGTCTTTACCCTGA
57	AGCTTTCCGGCACCTAAACGTTAATATTTAAACAGTTCAGAA
58	GGACGACGACAGTAAAATCAGCTCATTTCGGAATCGTCATAA
59	TGTAGATGGGCGCAAATTCGCGTCTGGCGGGTAATAGTAAAA
60	GAACAAACGGCGGAAAATGTGAGCGAGTCAAAATAGCGAGAG
61	CAGGGATAGCAAGCTTCGTCACCAGTACCAACACTATCATAA
62	CACCCTCAGAACCGACAGCCCTCATAGTCAGATACATAACGC
63	CACCGTACTCAGGATTTCCAGACGTTAGGATTCATCAGTTGA
64	CGTCGAGAGGGTTGTAAACAACTTTCAATAATAAAACGAACT
65	TAGGATTAGCGGGGAACAACTAAAGGAAGCTCATTATACCAG
66	ACATGAAAGTATTAAATCTCCAAAAAAAACTTTAATCATTGT
67	CAGTTAATGCCCCCCGGTTTATCAGCTTCCAGAACGAGTAGT
68	TTTTAACGGGGTCATGATACCGATAGTTAGCTGCTCATTCAG
69	CATACATGGCTTTTACGCATAACCGATATTGACAAGAACCGG
70	AATGGAAAGCGCAGTAAAGGCCGCTTTTAGACCAGGCGCATA
71	GGCCTTGATATTCAACAGCATCGGAACGCCGAACTGACCAAC
72	CGCCGCCAGCATTGGGACTAAAGACTTTTACTTAGCCGGAAC
73	AGCCACCACCCTCAAAAATACGTAATGCTCGCCTGATAAATT
74	CCTTCACCGCCTGGTGCAACAGTGCCACAACAGTTGAAAGGA
75	CGCTGGTTTGCCCCAGAAGATAAAACAGAGGAGCACTAACAA
76	TTCCGAAATCGGCACCCTAAAACATCGCTACATTTGAGGATT
77	CGAGATAGGGTTGATATTTTTGAATGGCGACAACTCGTATTA
78	ACTATTAAAGAACGACCCTTCTGACCTGAAAAGTTTGAGTAA
79	CGTCTATCAGGGCGACGACCAGTAATAAACCAGAAGGAGCGG
80	ATCAAGTTTTTTGGCGTCTGAAATGGATTGATGGCAATTCAT
81	GAACCCTAAAGGGAAGGAAAAACGCTCACTTCTGAATAATGG
82	GCCGGCGAACGTGGGCTGGTAATATCCAATTTGCACGTAAAA
83	AGCGGGCGCTAGGGAACATCACTTGCCTAGGTTTAACGTCAG
84	CGTAACCACCACACACGCAAATTAACCGATCGGGAGAAACAA
85	CGCGTACTATGGTTTTTATAATCAGTGACCAAGTTACAAAAT
86	CCTCGTTAGAATCAGATTTTAGACAGGATACCTGAGCAAAAG
87	TAAGAGCAAGAAACAGAGAGATAACCCAAAAATTAATTACAT
88	AGCCCTTTTTAAGAAACACCCTGAACAATAATGGAAACAGTA
89	CCAGAAGGAAACCGAACATAAAAACAGGCTTGCTTCTGTAAA

90	AAAGAACTGGCATGTTTAACGTCAAAAAAATCCTTGAAAACA
91	AGCAAACGTAGAAAGCCATATTATTTATAAGAGTCAATAGTG
92	AAGAAACGCAAAGAAGCGTCTTTCCAGACTACCTTTTTAACC
93	ATCAATAGAAAATTCACCCAGCTACAATATGTAAATGCTGAT
94	AGGGCGACATTCAAGGGAGGTTTTGAAGGAGAAAACTTTTTC
95	ACGGAAATTATTCAATTCTAAGAACGCGGACCTAAATTTAAT
96	TTGAGCCATTTGGGCGCCCAATAGCAAGAAGGCGTTAAATAA
97	ACCATTACCATTAGACAAGCAAGCCGTTAAAAAGCCTGTTTA
98	ATCGATAGCAGCACTTCCAAGAACGGGTCAGTATAAAGCCAA
99	GCCTTTAGCGTCAGGCATGTAGAAACCACGCCATATTTAACA
100	GGTCATAGCCCCCTAAGTCCTGAACAAGTTTTCGAGCCAGTA
101	GCAAATGAAAAATCACCAGTGAGACGGGTCGGCCAACGCGCG
102	GTATTAACACCGCCCCCTGAGAGAGTTGCTTTCCAGTCGGGA
103	AACGAACCACCAGCAGCAGGCGAAAATCGAGTGAGCTAACTC
104	GCGCGAACTGATAGAAATCCCTTATAAATACGAGCCGGAAGC
105	CGTGGCACAGACAAGTGTTGTTCCAGTTTTCCTGTGTGAAAT
106	CCAACAGAGATAGATGGACTCCAACGTCCCCGGGTACCGAGC
107	GATTCACCAGTCACATGGCCCACTACGTCAGTGCCAAGCTTG
108	TTTTGACGCTCAATGGTCGAGGTGCCGTAACGCCAGGGTTTT
109	CCAGCCATTGCAACGCCCCCGATTTAGAGCTGGCGAAAGGGG
110	AAACTATCGGCCTTCGAGAAAGGAAGGGTTGGGAAGGGCGAT
111	CTTTGATTAGTAATCGCTGGCAAGTGTAAAACCAGGCAAAGC
112	AGAGTCTGTCCATCCCGCCGCGCTTAATATCGCACTCCAGCC
113	TCCTGAGAAGTGTTGCTTTGACGAGCACCTGCCAGTTTGAGG
114	AGGCCGATTAAAGGGAGCGGGAGCTAAAATAGGTCACGTTGG
115	TGAGCGCTAATATCAATGAAATAGCAATTGGATTCTCCGTGG
116	GGGAGAATTAACTGAAAGTAAGCAGATAACCACCCTCATTTT
117	TTTACAGAGAGAATAGGAAACGCAATAAACCCTCAGAACCGC
118	GAAACGATTTTTTGATTAAGACTCCTTACGGAATAGGTGTAT
119	TTACAAAATAAACAATACATACATAAAGAGGCGGATAAGTGC
120	TACCAACGCTAACGCACCACGGAATAAGCTCAAGAGAAGGAT
121	TAGTTGCTATTTTGCATATGGTTTACCACCTATTATTCTGAA
122	ACCTCCCGACTTGCCCGATTGAGGGAGGAGTGCCCGTATAAA
123	AAGGCTTATCCGGTTTAAAGGTGAATTAGTACTGGTAATAAG
124	AGGAATCATTACCGAATTAGAGCCAGCAGTTCCAGTAAGCGT
125	CGCACTCATCGAGACAAGGCCGGAAACGCTCATTAAAGCCAG
126	GTCTTTCCTTATCACGTAATCAGTAGCGCAGGTCAGACGATT
127	ATCCTAATTTACGAACTGTAGCGCGTTTACCACCACCAGAGC
128	TATCAACAATAGATTATTAGCGTTTGCCACCGCCACCCTCAG
129	CAATCAATATCTGGATCAATTCTACTAACAAGGCAAAGAATT
130	ATTGAGGAAGGTTATTTAGCTATATTTTCATAAAGCTAAATC
131	CTAATAGATTAGAGAGTTTGACCATTAGAATACTTTTGCGGG
132	TAGAAGTATTAGACCCATATAACAGTTGAAATTTTTAGAACC
133	AATCCTTTGCCCGATTTTAAATATGCAATAATGTGTAGGTAA
134	CATTATCATTTTGCCTTAGAGCTTAATTACAGTCAAATCACC

135 AATTATCATCATATCTTTAATTGCTCCTGATAAATTAATGCC
136 CAATATAATCCTGACAGACCGGAAGCAAACAAAGGCTATCAG
137 AAGGGTTAGAACCTAGACTTCAAATATCAGAGAATCGATGAA
138 CAGAAATAAAGAAAAGCAAAGCGGATTGATATGTACCCCGGT
139 ATGAATATACAGTATAAATCAAAAATCAAAGATTGTATAAGC
140 TAACGGATTCGCCTCCCCTCAAATGCTTTTGTTAAAATTCGC
141 CGCGCAGAGGCGAAAGCGTCCAATACTGTTTAACCAATAGGA
142 AAGATGATGAAACAAGTTTTGCCAGAGGCTTCCTGTAGCCAG
143 TTAACAATTTCATTACGACGATAAAAACAACAACCCGTCGTA
144 CATAAATCAATATAAGGCATAGTAAGAGAAACTACAACGCCT
145 TCGTCGCTATTAATACATTCAACTAATGTAGCGTAACGATCT
146 TAGCGATAGCTTAGATTACAGGTAGAAATAAATGAATTTTCT
147 AATTTATCAAAATCAGAAAAATCTACGTCAGTTTCAGCGGAG
148 TCCGGCTTAGGTTGGATTTTAAGAACTGTTGCGAATAATAAT
149 GCAAATCCAATCGCATGGTTTAATTTCAAGGCTCCAAAAGGA
150 AAATATATTTTAGTCCTGACGAGAAACAGCTTTCGAGGTGAA
151 GGTTTGAAATACCGAATCAACGTAACAAGCGCCGACAATGAC
152 GAATAAACACCGGACATCAAGAGTAATCTATTCGGTCGCTGA
153 GTATCATATGCGTTGATGAACGGTGTACGCGGGATCGTCACC
154 CGCTCAACAGTAGGCAATCATAAGGGAAAGGGTAGCAACGGC
155 ACGCCAACATGTAAGACCTGCTCCATGTTTCATGAGGAAGTT
156 ATAAGAGAATATAAGGAGATTTGTATCACACTACGAAGGCAC
157 CAATAAATCATACAGGTAGTAGTAGCATTATC
158 GGGAGAGGCGGTTTGCGTATTGG
159 GCGCCAGGGTGGTTTTTCTTTTCTAAAGCATCACCTTGCTGA
160 ACCTCAAATATCAAACCCT
161 CTTTGACCCCCAGCGAACACTAAAACACTCAT
162 AGAGGCAAAAGAATTTATACCAAGCGCG
163 CACCGGAACCGCCTCCCTCAGAG
164 AAAATCACCGGAACTGTTCAGCTAATGC
165 CAATAAACAACACAGAGCCAC
166 GTAAAGTAATTCTGTCCAGACGACGA
167 AAAAAAAAAAAAAAAAAAAAACAAAGTACAACAGTACCG
168 AAAAAAAAAAAAAAAAAAACAAAAGAGAACGCGCCTGTT
169 AAAAAAAAAAAAAAAAAAACAATTCTATTAGTCTTTAAT
170 AAAAAAAAAAAAAAAAAAATCTTCTAGGCGTTTTAGCGA
171 AAAAAAAAAAAAAAAAAACAAAAGGAATTACGTGTGAGT
172 AAAAAAAAAAAAAAAAAACTATTATAGTCAGATTGCGTA
173 AAAAAAAAAAAAAAAAAACTGGAAGTTTCATTTTTACAA
174 AAAAAAAAAAAAAAAAAAGAATAACGAAGCGCATTAGAC
175 AAAAAAAAAAAAAAAAAAGATTTTCGAGTAGAAGAACTC
176 AAAAAAAAAAAAAAAAAAGCAAATCGCTGAGAGCCAGCA
177 AAAAAAAAAAAAAAAAAAGCTGAAAAGGTGGCTCAGTTG
178 AAAAAAAAAAAAAAAAAAGGATTAGAGAGTACTCCTGAT
179 AAAAAAAAAAAAAAAAAATATCAGATATTTACATTGGCA

180 AAAAAAAAAAAAAAAAAATCAGGACGTTGGGAATAGGTC
181 AAAAAAAAAAAAAAAAAATGAATAAGGCTTGCTAATTTC
182 AAAAAAAAAAAAAAAAAATGAGAGAGCCTAATTTGCCAG
183 AAAAAAAAAAAAAAAAAATGTTTAGACTGGATTTATTCA
184 AAAAAAAAAAAAAAAAAATTCTTACATTAAACCAAGTAC
185 AAAAAAAAAAAAAAAAAATTTCAATACGGTACGCCAGAA
186 AAAAAAAAAAAAAAAAAATTTGAAAGAGGACAATACAAA
